# Perioperative Blood Transfusion and Infectious Complications Among Pediatric Patients with Cancer

**DOI:** 10.3390/children12020160

**Published:** 2025-01-29

**Authors:** Elizabeth D. Cochran, Jillian C. Jacobson, Arti Machchhar, Jingbo Qiao, Dai H. Chung

**Affiliations:** 1 Department of Pediatric Surgery, University of Texas Southwestern Medical Center, Dallas, TX 75390, USA; elizabeth.cochran@utsouthwestern.edu (E.D.C.); jillian.jacobson@utsouthwestern.edu (J.C.J.); arti.machchhar@utsouthwestern.edu (A.M.); jingbo.qiao@utsouthwestern.edu (J.Q.); 2 Children’s Health, Dallas, TX 75235, USA

**Keywords:** transfusion, immunomodulation, infection, pediatric cancer, operative complications

## Abstract

**Background:** Perioperative blood transfusion has been identified as a risk factor for postoperative infectious complications in adult patients with cancer. This study aimed to determine whether this association also exists in pediatric patients with cancer. **Methods:** A retrospective analysis was performed using the American College of Surgeons National Surgical Quality Improvement Program Pediatric (NSQIP-P) database. Pediatric patients with an active cancer diagnosis at the time of surgical intervention from 2015 to 2019 were reviewed. Statistical analysis was performed using Pearson chi-square and Fisher’s exact tests as well as multiple logistic regression. **Result:** In total, 14,973 pediatric patients who underwent a surgical procedure and had an active cancer diagnosis at the time of operation were identified. Of these, 2602 patients (17.4%) received a perioperative blood transfusion (PBT). Patients who received a PBT experienced higher rates of postoperative infectious complications, including surgical-site infection (*p* < 0.0001), pneumonia (*p* < 0.0001), urinary tract infection (*p* < 0.0001), *C. difficile* infection (*p* < 0.0001), central-line-associated bloodstream infection (*p* < 0.0001), and sepsis (*p* < 0.0001). Patients who received a PBT also had increased 30-day mortality compared with those who did not receive a PBT (*p* < 0.0001). On multivariate analysis, PBT remained an independent risk factor for postoperative infectious complications (OR 1.9, 95% CI 1.61–2.32) and death (OR 1.8, 95% CI 1.23–2.71). **Conclusions:** Pediatric patients with cancer who undergo surgery and receive a blood transfusion in the perioperative period have increased 30-day mortality and are at increased risk for postoperative infectious complications. Considering that these patients are often immunosuppressed at baseline, infections can be particularly devastating in this population. As such, it is important to carefully consider the risks and benefits of PBT prior to transfusion.

## 1. Introduction

Blood transfusions play an integral role in modern surgical care. The use of transfusions to minimize the sequelae of intraoperative blood loss, or to decrease the volume of blood lost through the transfusion of plasma containing clotting factors, has allowed surgeons to provide more aggressive operative interventions that would previously have been considered too risky to attempt. This is particularly relevant in the surgical treatment of patients with cancer, who often require complex resections of highly vascular tumors and who are frequently pancytopenic at baseline due to treatment or disease-related myelosuppression. While the benefits of blood transfusion are clear and, often lifesaving, the transfusion of blood products is not free of risk. Although frequently an area of patient concern, the risk of transmission of blood-borne infections through transfused blood is very low, less than one in one million per unit transfused in the United States [1]. However, though the transfused blood itself is unlikely to be a source of infection, increasing evidence suggests that the receipt of a blood transfusion triggers significant immunomodulatory changes. These changes have been implicated in the increased rate of postoperative infections seen among transfused patients in multiple surgical populations [2,3,4,5,6,7].

The mechanisms by which transfusion-related immunomodulation (TRIM) occurs remain poorly understood; however, the predominant theories focus on the presence of residual white blood cells (WBCs) found in packed red blood cells (PRBCs), even following leukoreduction, as well as the role of soluble extracellular immune mediators that build up in the PRBCs due to the storage- and age-related damage and degradation of cells [8]. Following transfusion, the presence of these immune mediators is believed to produce a transient immunosuppressive state by increasing anti-inflammatory cytokine release, decreasing macrophage cytokine production, and impairing neutrophil and T-cell function. It has been noted in several studies that, in critically ill children, transfusion-related immunosuppression appears to be most pronounced following the transfusion of RBCs with a longer storage time [9,10,11]. In addition to its impact on postoperative infections, the clinical implications of the transfusion-related immunosuppressive state are varied: it has been credited with improved graft survival in patients following kidney transplant but has also been associated with an increased risk of cancer recurrence and cancer-related death among adult patients with colorectal, gastric, and head and neck cancer [12,13,14,15].

The complex interplay between the immune system and the development and treatment of cancer makes the role of TRIM particularly important to consider in the perioperative management of patients with cancer. These patients are at increased risk for TRIM-related complications for multiple reasons, including the common need for operative intervention as a part of cancer treatment, an increased likelihood of receiving a perioperative transfusion due to baseline anemia, and significant vulnerability to infection due to disease- and treatment-related immunosuppression. While the association between perioperative blood transfusion and postoperative infections has been well established in adult patients with cancer, there are scarce data available regarding pediatric patients with cancer [13,16,17]. Historically, there has been a trend toward a less judicious use of blood transfusions in pediatric populations, but recent studies have indicated that this may not be necessary and may even be harmful, particularly in children with cancer [18,19]. This study aims to determine whether perioperative blood transfusion is associated with an increased risk for postoperative infectious complications and 30-day mortality among children with cancer.

## 2. Materials and Methods

A retrospective analysis was performed using the American College of Surgeons National Surgical Quality Improvement Program Pediatric (NSQIP-P) database. Pediatric patients (age < 19 years) with an active cancer diagnosis at the time of any surgical intervention from 2015 to 2019 were reviewed. The patients were separated into two groups based on whether they received a red blood cell (RBC) transfusion during the perioperative period (defined as 48 h before surgery through 72 h following surgery). Those who received a transfusion were then further divided into two groups based on whether they received blood preoperatively or intra- and/or postoperatively. Analyses were performed treating transfusion as a categorical variable, comparing those who received any RBC transfusion and those who received no RBC transfusion, as well as a continuous variable, to facilitate the assessment of the impact of total transfusion volume. We compared demographics, patient characteristics, operative characteristics, and outcomes between groups. Outcomes of interest were the development of postoperative infectious complications and 30-day mortality. Infectious complications were defined as surgical-site infection (SSI), pneumonia, urinary tract infection (UTI), sepsis, septic shock, central-line-associated bloodstream infection (CLABSI), and *C. difficile* infection within 30 days of operative intervention. Statistical analysis was performed using Pearson chi-square, Fisher’s exact test, ANOVA, and multiple logistic regression, as appropriate. Variables included in the regression analyses included patient demographics (age, sex, race), preoperative clinical characteristics (ASA class; cardiac history; receipt of steroids; known preoperative infections; nutritional support requirement; hematologic disorders; need for CPR or inotropic support; and preoperative lab work including white blood cell count, hematocrit, platelets, PT, INR, and PTT), and operative characteristics (minimally invasive versus open procedure, urgency of case, inpatient or outpatient procedure, wound class, and anesthesia time). A *p* value of <0.05 was considered statistically significant. All analyses were performed using GraphPad Prism Version 10 (GraphPad Software, La Jolla, CA, USA).

## 3. Results

### 3.1. Risk Factors for Blood Transfusion

**Figure 1:**
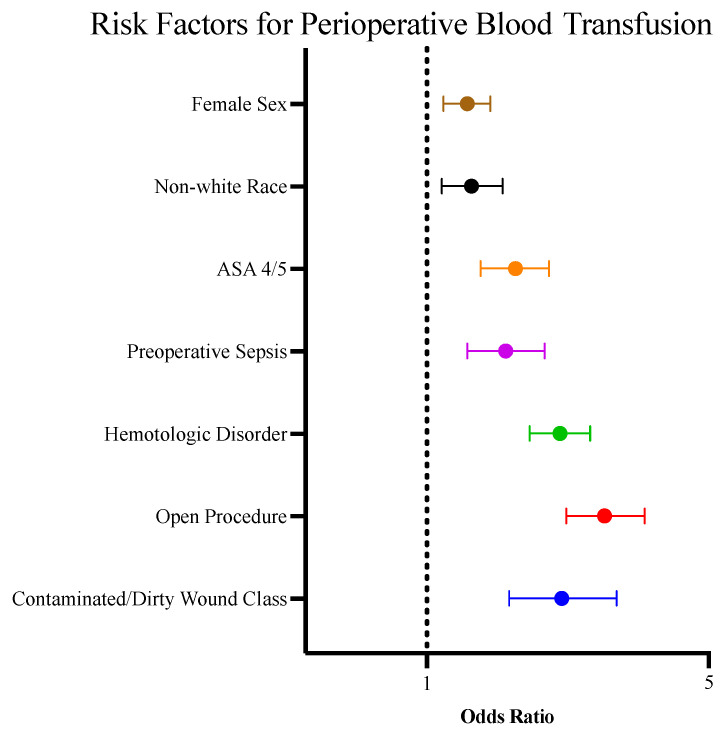
Forest plot depicting patient and operative characteristics associated with increased risk for perioperative blood transfusion. The reference categories for each risk factor above are as follows: female sex vs. male sex, non-white race vs. white race, ASA 4/5 vs. ASA 1/2/3, preoperative sepsis vs. no preoperative sepsis, hematologic disorder vs. no hematologic disorder, open procedure vs. entirely minimally invasive procedure, contaminated/dirty wound class vs. clean or clean/contaminated wound class.

All 14,973 pediatric patients who underwent a surgical procedure from 2015 to 2019 and had an active cancer diagnosis at the time of operation were identified. Of these, 2602 patients received a perioperative blood transfusion, with 627 receiving blood preoperatively and 1975 receiving blood intra- or postoperatively. The average age of the patients who received blood was 5.9 years (vs. 9.4 years, *p* < 0.0001). On multivariable analysis, patients who received transfusions were more likely to be female and of non-white race (*p* = 0.0009 and *p* = 0.0031, respectively) (Figure 1). The clinical characteristics associated with an increased risk of transfusion included the presence of known hematologic disorder, preoperative sepsis, and an American Society of Anesthesiologists (ASA) score of 4 or above (all *p* < 0.0001). An increased preoperative white blood cell count and a decreased preoperative hematocrit also carried an increased risk for blood transfusion (*p* = 0.0183 and *p* < 0.0001, respectively). The most common procedures associated with the receipt of a perioperative blood transfusion were craniectomy (n = 607), nephrectomy (n = 380), and hepatectomy (n = 209). The operative characteristics associated with an increased risk for transfusion were the utilization of an open approach and contaminated/dirty wound class (both *p* < 0.0001).

### 3.2. Infectious Complications Associated with Perioperative Blood Transfusion

**Figure 2:**
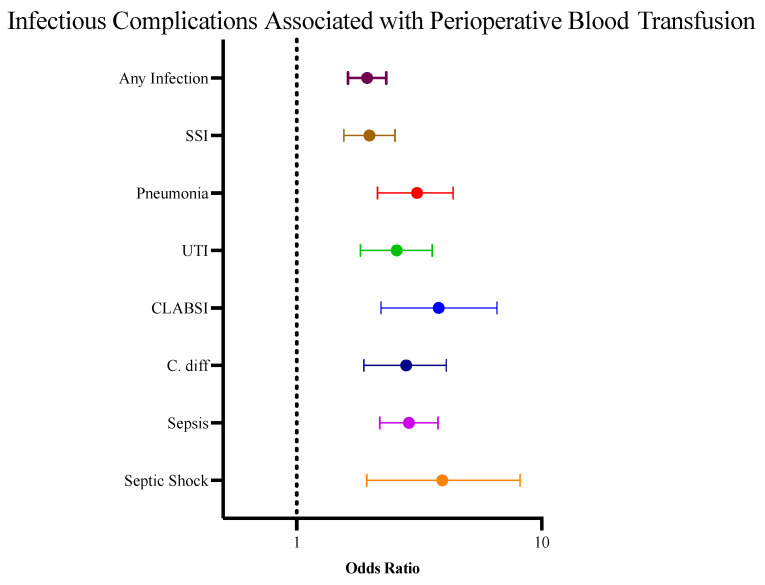
Forest plot depicting the increased risk of postoperative infectious complications associated with receipt of perioperative blood transfusions. The reference categories for each risk factor above are as follows: any infection vs. no infection, SSI vs. no SSI, pneumonia vs. no pneumonia, UTI vs. no UTI, CLABSI vs. no CLABSI, C. diff vs. no C. diff, sepsis vs. no sepsis, septic shock vs. no septic shock.

Overall, 5.0% of patients experienced a postoperative infectious complication with an increased prevalence among those who received a perioperative blood transfusion (9.68% vs. 4.07%, *p* < 0.0001). On multivariable analysis controlling for potential confounders, which included patient comorbidities, preoperative sepsis, wound infection, steroid use, nutritional support requirement, ASA class, wound class, case urgency, and operative approach, the receipt of a blood transfusion during the perioperative period remained an independent predictor of postoperative infectious complications (OR 1.654, 95% CI 1.223–2.232) (Figure 2). Blood transfusion had a dose-dependent impact on postoperative infection risk, with the rate of infectious complications increasing by 14.5% for every 10 mL/kg of blood transfused (OR 1.145, 95% CI 1.09–1.207). On univariable analysis, patients who were transfused prior to surgery had an increased risk of septic shock compared with those who only received blood intra- or postoperatively (*p* = 0.0002). However, no association between timing and overall risk of infectious complication was seen in the multivariable analysis.

### 3.3. Increased Mortality Associated with Perioperative Blood Transfusion

**Figure 3:**
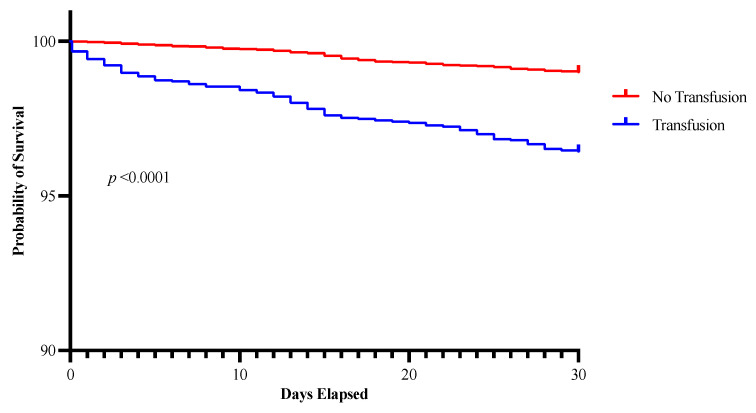
Kaplan–Meier curve depicting the association between perioperative blood transfusion and an increased risk of death in the 30 days following surgery.

The overall 30-day mortality was low at 1.46%. However, the risk of death was increased among patients who received a perioperative blood transfusion (*p* < 0.0001) (Figure 3). Transfusion remained an independent predictor of 30-day mortality on multivariable analysis, with patients who received a perioperative blood transfusion experiencing a 1.83 times higher risk of death than those who did not receive blood (95% CI 1.24–2.71). Blood transfusion had a dose-dependent impact on 30-day mortality, with the risk of death increasing by 3.2% for every 10 mL/kg of blood transfused (OR 1.032, 95% CI 1.003–1.074). On univariable analysis, patients who were transfused prior to surgery had an increased risk of death compared with those who only received blood intra- or postoperatively (*p* < 0.0001); however, this did not persist on multivariable analysis.

## 4. Discussion

In this study, 17.3% of pediatric patients with cancer received a perioperative blood transfusion compared with a transfusion rate of 1.5% reported for pediatric surgical patients in general [20]. The risk factors for transfusion identified here, young age and preoperative severe illness, were consistent with those in prior studies focused on transfusion utilization in pediatric surgical patients [21]. Little has been published regarding perioperative transfusion thresholds in the pediatric cancer population. However, current recommendations suggest a hemoglobin transfusion threshold of 7–8 g/dL for pediatric oncologic patients in general [22]. Here, we found that patients who received a blood transfusion had a mean hematocrit of 30.47%, corresponding to a hemoglobin of approximately 10 g/dL, suggesting that a more liberal transfusion approach may be being utilized for pediatric patients with cancer during the perioperative period. This also begs the question of whether transfusion and any related complications may have been avoidable in a subset of these patients if a lower transfusion threshold, in keeping with the current recommendations, was used. That said, the hematocrit levels reported in the NSQIP-P database are all preoperative, so it is possible that intra- and postoperative transfusions were triggered by lower levels identified by later lab draws that were not included in the NSQIP-P data. Overall, there is a paucity of data available regarding optimal transfusion thresholds for pediatric patients with cancer in the perioperative setting, and this should be the focus of future research.

We found a dose-dependent association between perioperative blood transfusion and postoperative infectious complications. This association has been well established in adult populations but is variable in the small number of studies focused on this issue in the pediatric cancer population. In their analyses of perioperative blood transfusion among pediatric patients with solid tumors undergoing tumor resection or biopsy, Acker et al. report an increased rate of infectious complications associated with perioperative blood transfusion, while Gonzalez et al. found no association [21,23]. Both studies only included patients with solid tumors and transfusions related to tumor resection or biopsy. As pediatric patients with cancer with both solid and hematologic malignancies frequently undergo surgical procedures outside of resection or biopsy, we sought to expand the range of diagnoses and procedures included in our analysis. In doing so, we found that at least 10% of the patients who received a perioperative transfusion and developed a postoperative infectious complication did so when undergoing a non-oncologic operation, suggesting that even minor procedures may lead to blood transfusion and associated infectious complications in pediatric patients with cancer, and analyses considering only tumor resections or biopsies may be too narrow in scope to fully evaluate the risks of perioperative transfusion in the pediatric cancer population.

Finally, our study identified a dose-dependent association between perioperative blood transfusion and 30-day mortality in pediatric patients with cancer undergoing surgery. To our knowledge, this is a novel finding. The available data on this subject are sparse and have focused primarily on cancer recurrence and cancer-related mortality, and the reported results are variable. A 2023 study by Acker et al. reported an increased rate of cancer recurrence but no increased mortality among pediatric patients with solid tumors who received a perioperative blood transfusion during or following tumor resection [23]. Meanwhile, Muller et al. reported in their 2022 study that perioperative blood transfusion was associated with decreased recurrence-free survival but unchanged overall survival among children undergoing nephroblastoma resection [24]. Meanwhile, Owusu-Agyemang et al. found no association between perioperative transfusion and recurrence or survival among pediatric patients with cancer undergoing cytoreductive surgery and hyperthermic intraperitoneal chemotherapy [25]. We are aware of only one other study analyzing the impact of transfusion on 30-day mortality in pediatric patients with cancer, which found no association between perioperative transfusion and 30-day mortality among children undergoing biopsy or resection of solid tumors [22]. However, a 2016 study by Goobie et al. utilizing the NSQIP-P database found a dose-dependent increase in 30-day mortality and infectious complications among children who received perioperative blood transfusions when undergoing noncardiac surgery [26]. Similar to our study, Goobie’s analysis included a broader patient base and a wider range of included procedures than the other studies, again suggesting that analyzing the impact of transfusion only in the setting of major oncologic operations may lead to missed complications.

After acknowledging that perioperative blood transfusion is associated with postoperative complications in pediatric patients with cancer, the question then becomes how to minimize transfusion requirements in this patient population. Patient blood management (PBM), a multidisciplinary approach to optimizing and preserving a patient’s own blood volume in the pre- and intraoperative settings in order to minimize exposure to blood products, has been increasingly incorporated in the perioperative care of pediatric patients [27]. In the preoperative setting, the early identification of iron deficiency and treatment with iron supplements or erythropoietin (EPO) have been shown to improve preoperative hemoglobin levels and decrease perioperative transfusion requirements in some pediatric surgical populations [28]. Meanwhile, the minimization of intraoperative blood loss through the utilization of cell saver salvaged blood has been found in randomized trials to decrease postoperative blood transfusions in pediatric patients undergoing cardiac surgery [29]. Furthermore, the implementation of PBM practices has been associated with reductions in inappropriate transfusions and postoperative complications in adult patients with cancer [30,31]. However, despite these promising findings, there are scarce data available assessing the utilization and impact of PBM practices in pediatric patients with cancer.

The limitations of this study include the inherent limitations of retrospective analysis, as well as the lack of granularity associated with use of large retrospective databases. Although the use of the NSQIP-P database provides information on a large number of patients, it does not allow access to data regarding the operative and perioperative clinical conditions that likely influenced decision making and patient outcomes. While certain markers for these conditions can be utilized, like preoperative lab results as an indicator of preoperative infection or compromised immune status, these findings are impacted by the cancer and cancer treatments, making them less reliable in this patient population.

## 5. Conclusions

It is well established that, while blood transfusions can be lifesaving, they also have important immunomodulatory effects that have a significant impact on postoperative outcomes, particularly regarding infection. This is of increased concern when operating on patients with cancer, who are at an increased risk of both requiring blood transfusion and developing serious infectious complications. Our study found that, among children with cancer undergoing surgery, red blood cell transfusion in the perioperative period is associated with a dose-dependent increase in the risk of postoperative infectious complications and 30-day mortality. Furthermore, we found that the timing of the transfusion in relation to surgery impacts the risk of transfusion-related complications, with preoperative transfusions being associated with an increased risk of death compared with intra- or postoperative transfusions, suggesting that the common practice of prophylactic transfusion of pediatric patients with cancer undergoing surgery should be re-evaluated, and surgeons should consider placing blood on hold rather than transfusing preoperatively. These risks should be carefully considered when deciding when to transfuse, and efforts should be made to minimize the total volume of blood received. Finally, future studies should investigate the potential role of patient blood management practices in minimizing perioperative blood transfusions in pediatric patients with cancer.

## Data Availability

The data that support these findings are housed with the American College of Surgeons. Data are available in a de-identified fashion to participants of the NSQIP-Pediatrics program.
